# Editorial: Integrating ketogenic diet with physical exercise: implications for athletes and chronic conditions

**DOI:** 10.3389/fnut.2026.1785548

**Published:** 2026-01-27

**Authors:** Roberto Cannataro, Diego A. Bonilla, Maria Cristina Caroleo, Giuseppe Cerullo, Richard B. Kreider, Erika Cione

**Affiliations:** 1 Department of Biology, Ecology and Earth Sciences, University of Calabria, Rende, Italy; 2 Research Group in Sports Nutrition (DBSS-Nut), Dynamical Business & Science Society—DBSS International SAS, Bogotá, Colombia; 3 Hologenomiks Research Group, Department of Genetics, Physical Anthropology and Animal Physiology, University of the Basque Country (UPV/EHU), Leioa, Spain; 4 Grupo de Investigación NUTRAL, Facultad Ciencias Alimentarias y Farmacéuticas, Universidad CES, Medellín, Colombia; 5 Department of Health Sciences, University of Magna Graecia Catanzaro, Catanzaro, Italy; 6 Department of Biomedical Sciences, University of Padua, Padua, Italy; 7 Exercise & Sport Nutrition Lab, Department of Health & Kinesiology, Texas A&M University College Station, Killeen, TX, United States; 8 Department of Pharmacy, Health and Nutritional Sciences, University of Calabria, Rende, Italy

**Keywords:** athletes, ketogenic diet, ketosis, mitochondrial biogenesis, physical exercise

Studies on the ketogenic diet date back almost 100 years, but most studies are no older than 15 years, demonstrating that this nutritional plan has recently received significant attention from the scientific community; the ketogenic diet is a program that usually allows no more than 20–30 g of carbohydrates per day or 5% of the overall caloric intake; in this condition, ketosis is triggered. The ratio of lipids to proteins can vary depending on the specific goals of the diet (1).

In recent years, ketogenic diets have been used to treat conditions such as epilepsy as well as to support chemotherapy (2) or chronic conditions such as lipedema (3). In this instances, the ketogenic diet seems to be effective (4).

In treating health conditions, there is also a need to combine the diet with physical activity; physical activity is important for achieving long-term results, especially if losing weight is the final goal (5).

We have shown that exercise, particularly with resistance training, has a significant impact on mitochondrial biogenesis, a key aspect of the ketogenic diet (6).

Findings on athletic performance appear inconsistent (7). Furthermore, there is a scarcity of research concerning pathological conditions, highlighting the need for more data; caloric intake likely represents the key variable (8).Using animal models, Kim et al. have shown that a ketogenic diet combined with aerobic exercise results in an activation of mitochondrial biogenesis, lipolysis, and thermogenesis, resulting in a browning of white adipose tissue (WAT).

The case report presented by Russell and Schwartz highlights the different recruitment of energy substrates based on exercise intensity. This confirms what we see in high-level sports practice: the ability to maintain ketosis even with a carbohydrate intake higher than the typical 20–30 g per day. This is an important indication of the effectiveness of the synergy between a ketogenic diet and exercise.

Carrera-Juliá's group demonstrated that short-chain fatty acid supplementation, specifically coconut oil, promoted moderate ketogenesis in patients with amyotrophic lateral sclerosis (ALS). This effect could be beneficial, as ketogenic diets could be effective in supporting this disease. However, compliance with a high fat intake can be poor, whereas with the support of MCTs, a more palatable protein-to-fat ratio could be used.

Finally, an important point is emphasized by Silva et al. regarding the evaluation of the results obtained, which is too often inconsistent; this manuscript highlights how even a method considered the gold standard can yield misleading results, especially in specific populations such as adolescent athletes.

The right combination of exercise and diet should always be considered, especially when considering a nutritional program that severely restricts a single nutrient However, this should not be a limiting factor in combining the two, as they work together to provide better, longer-lasting results.
